# Imported Episodic Rabies Increases Patient Demand for and Physician Delivery of Antirabies Prophylaxis

**DOI:** 10.1371/journal.pntd.0000723

**Published:** 2010-06-22

**Authors:** Zélie Lardon, Laurence Watier, Audrey Brunet, Claire Bernède, Maryvonne Goudal, Laurent Dacheux, Yolande Rotivel, Didier Guillemot, Hervé Bourhy

**Affiliations:** 1 INSERM, U657 and Institut Pasteur, PhEMI, Paris, France; 2 Institut Pasteur, Centre National de Référence pour la Rage, Paris, France; 3 École Nationale des Services Vétérinaires, Marcy l'Étoile, France; 4 Faculté de Médecine Paris Île de France-Ouest, Université de Versailles–Saint-Quentin, Versailles, France; Centers for Disease Control and Prevention, United States of America

## Abstract

**Background:**

Imported cases threaten rabies reemergence in rabies-free areas. During 2000–2005, five dog and one human rabies cases were imported into France, a rabies-free country since 2001. The Summer 2004 event led to unprecedented media warnings by the French Public Health Director. We investigated medical practice evolution following the official elimination of rabies in 2001; impact of subsequent episodic rabies importations and national newspaper coverage on demand for and delivery of antirabies prophylaxis; regular transmission of epidemiological developments within the French Antirabies Medical Center (ARMC) network; and ARMC discussions on indications of rabies post-exposure prophylaxis (RPEP).

**Methodology/Principal Findings:**

Annual data collected by the National Reference Center for Rabies NRCR (1989–2006) and the exhaustive database (2000–2005) of 56 ARMC were analyzed. Weekly numbers of patients consulting at ARMC and their RPEP- and antirabies-immunoglobulin (ARIG) prescription rates were determined. Autoregressive integrated moving-average modeling and regression with autocorrelated errors were applied to examine how 2000–2005 episodic rabies events and their related national newspaper coverage affected demand for and delivery of RPEP. A slight, continuous decline of rabies-dedicated public health facility attendance was observed from 2000 to 2004. Then, during the Summer 2004 event, patient consultations and RPEP and ARIG prescriptions increased by 84%, 19.7% and 43.4%, respectively. Moreover, elevated medical resource use persisted in 2005, despite communication efforts, without any secondary human or animal case.

**Conclusions:**

Our findings demonstrated appropriate responsiveness to reemerging rabies cases and effective newspaper reporting, as no secondary case occurred. However, the ensuing demand on medical resources had immediate and long-lasting effects on rabies-related public health resources and expenses. Henceforth, when facing such an event, decision-makers must anticipate the broad impact of their media communications to counter the emerging risk on maintaining an optimal public health organization and implement a post-crisis communication strategy.

## Author Summary

Rabies has been eliminated from a large part of the European Union and, thus, any newly imported cases threaten its reemergence. The 2000–2005 data derived from the exhaustive surveillance system implemented in France was analyzed to evaluate the impact on demand for and delivery of antirabies prophylaxis following introduction of five rabies-infected dogs and one infected human into this rabies-free area. Using these events, we were able to illustrate the difficulties encountered in reducing the demand for and prescription of post-exposure rabies prophylaxis in this context of episodic importation. Moreover, we highlighted the need for public health decision-makers to anticipate the broad spectrum of consequences of their media communications and to prepare appropriate responses (in terms of health resources) to maintain an optimally effective public health organization after importation of an exotic infectious agent or its emergence. These responses are particularly relevant in the context of limited availability of rabies post-exposure prophylaxis, especially antirabies immunoglobulin.

## Introduction

Media-communicated health alerts are being used more-and-more frequently by public health decision-makers to prevent consequences of a sudden event, such as, emerging and episodic zoonotic diseases. The medical community must now consider these communications to be preventive intervention tools for public health officials [1]–[3]. Obviously, as during any effective health intervention, undesired effects may also occur, such as rapidly rising numbers of potential cases to treat, leading, in turn, to health-resource saturation, especially if the pathogen involved is rare [4], [5].

Rabies is a viral encephalitis [6] that is considered to be a reemerging zoonosis throughout much of the world [7]. In Western Europe, rabies in non-flying terrestrial mammals was a well-known illness that has now become a rare disease, because many countries have succeeded in eradicating it. The major risk of rabies is now due to translocation of infected animals, mainly dogs, from rabies-enzootic areas and humans with rabies infection acquired abroad [8]. Although untreated rabies is invariably fatal, death can be avoided by proper administration of rabies post-exposure prophylaxis (RPEP), e.g., antirabies vaccine, with or without antirabies immunoglobulins (ARIG), before disease onset [6]. Thus, rapid identification of individuals potentially exposed to rabies is critical and media alerts can be extremely useful to identify people who were in contact with the rabid animal.

In France (60,000,000 inhabitants, 675,417 km^2^), primary health-care management of patients seeking RPEP is delivered through an official national network of Antirabies Medical Centers (ARMC), which are distributed throughout the country. RPEP is administered, predominantly according to the Zagreb schedule, to people bitten by an animal suspected of being infected with rabies or exposed to its saliva. Clinicians conduct a risk assessment for each exposed patient, and decide to administer RPEP according to the general recommendations, epidemiological data and grade of the bite [9]. The French network for rabies prophylaxis provides exhaustive national data collected by ARMC [10], and laboratory diagnoses of humans suspected of having rabies [11] and animals suspected contaminating humans. From 1968 to 1998, a period during which rabies was endemic in French foxes, more than 45,600 animals were diagnosed as rabid. In 2001, France was declared free of rabies in non-flying terrestrial mammals based on World Organisation for Animal Health (OIE) criteria and, as a consequence, the number of RPEP began to decline progressively.

However, in summer 2004, one imported rabid dog generated unprecedented media communications by the Public Health Director, whose official press release, dated 31 August 2004, warned, “At least, nine people are at risk of death and are actively and intensively being sought by the health authorities…” During this episode, antirabies vaccine stocks in ARMC were almost exhausted, leading to a temporary marketing license for the multidose Verorab vaccine (Sanofi Pasteur), which had not previously been authorized in France. That ARIG supplies were dangerously low is illustrated by the postponement of ARIG injections in some ARMC until day 7 after starting RPEP [12], [13] for several patients.

Controlling rabies reintroduction and communicating the risk of rabies spread remain a challenge to public health officials in rabies-free areas. In this study, we analyzed why and how the French rabies-control organization became so oversaturated. In particular, we examined the impact of newspaper reports on the numbers of patients consulting at ARMC, and their RPEP and ARIG prescriptions.

## Methods

### Ethics statement

This research has complied with the French national guidelines and Institut Pasteur policy. The analysis of data collected by the National Reference Center for Rabies (NRCR) from the AMRC was done anonymously and approved by the Commission Nationale Informatique et Liberté (Agreement #416031, dated 28 March 1996). This specific project was submitted to the Institut Pasteur Biomedical Research Committee (RBM/2006.025) and was approved on 19 December 2006.

### Data

**Figure 1:**
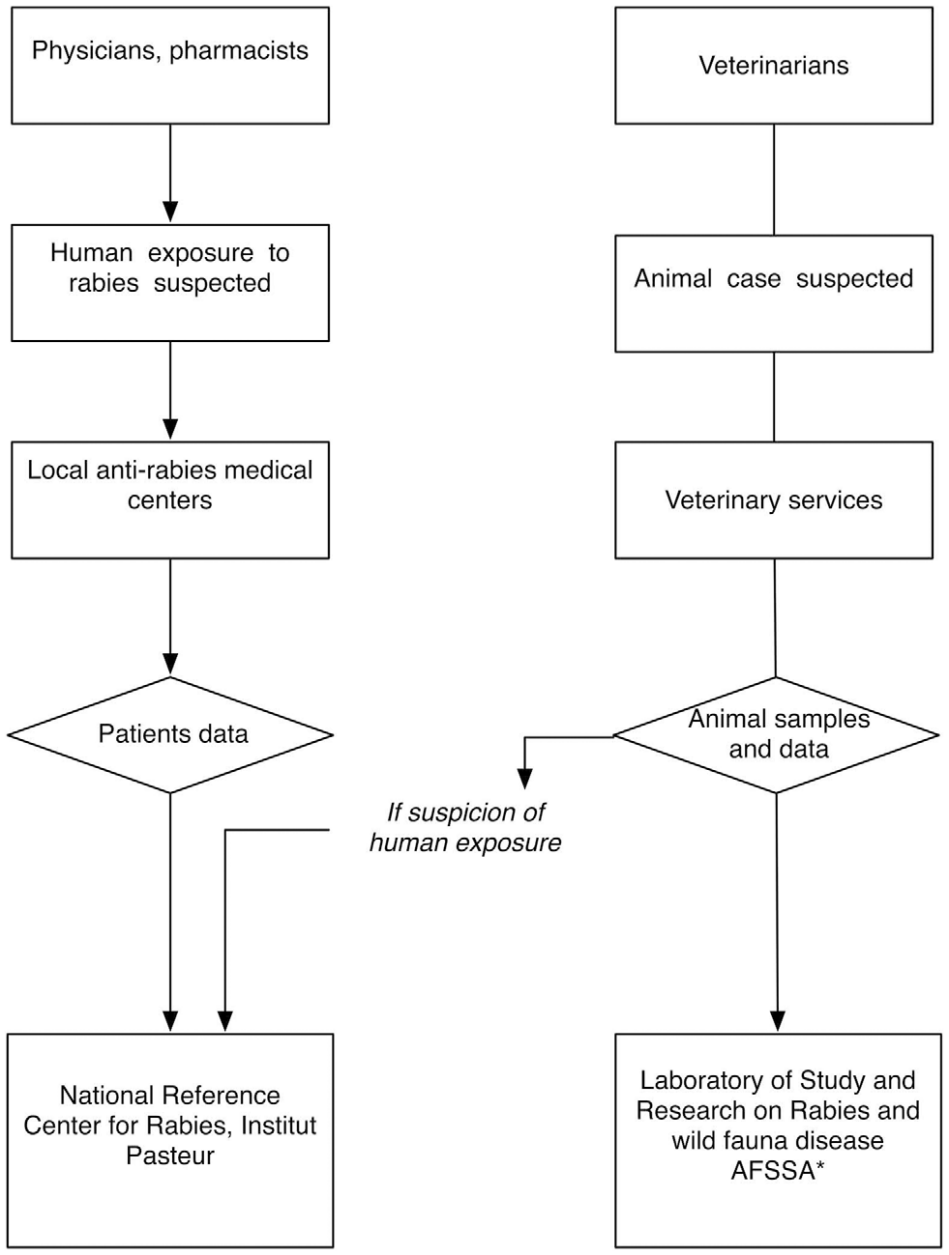
Flow chart of the French surveillance system for prevention of rabies in humans. *AFFSA denotes for French Agency for Food Safety, http://www.afssa.fr/.

**Figure 2:**
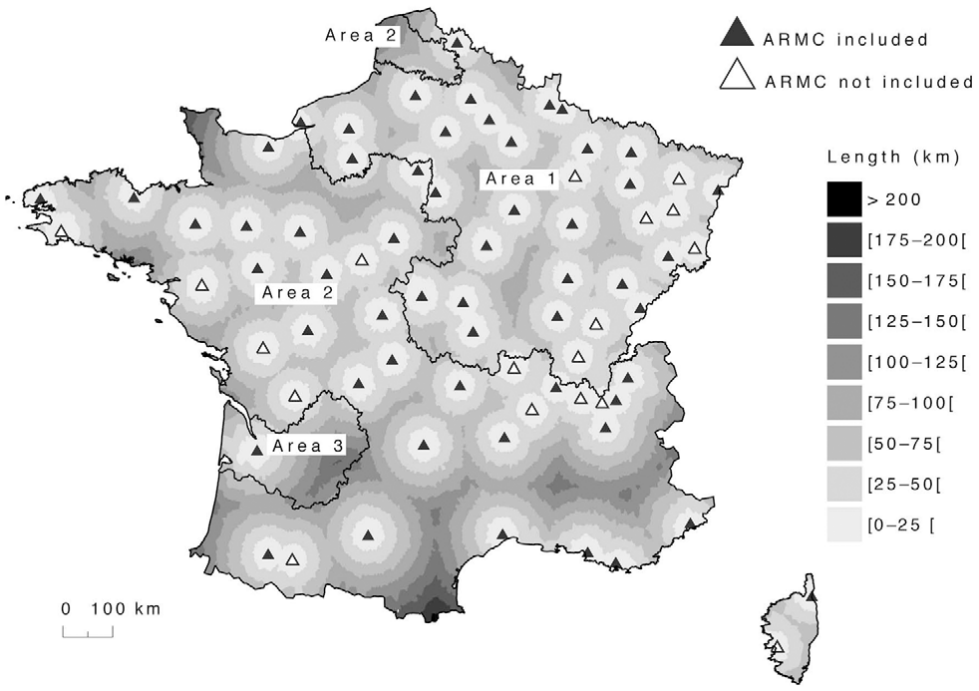
Spatial distribution of French AntiRabies Medical Centers (ARMC). The minimal distance to any of the 74 ARMC is illustrated (grey scale); 93% of the 36,539 districts are <75 km from an ARMC. France has been divided into three areas, according to their rabies experience: area 1 corresponds to the former zone harboring rabies-infected foxes (19,132,787 inhabitants); area 2, has no history of rabies events (37,423,439 inhabitants); and area 3, where rabies event #6 occurred (1,981,313 inhabitants).

French veterinary and human authorities work in close collaboration to detect cases and organize the medical responses to rabies (Figure 1), with a territorial network of 96 veterinary services and 74 ARMC disseminated throughout continental France, in 2004 (Figure 2). On the one hand, each animal responsible for human exposure is confined under veterinary surveillance. If dead and for whatever the reason, diagnostic laboratory tests are conducted at the NRCR, Institut Pasteur, Paris, France. On the other hand, ARMC are the only primary care centers allowed to prescribe RPEP. For each patient, a standard case-report form (Table S1) is systematically filled out describing important epidemiological features, such as geographic location, consultation date, type of exposure, animal species, contact date with the animal, medical decision concerning RPEP. Based on the data collected by ARMC, annual reports are written, which describe the patients visiting ARMC and those receiving RPEP (http://www.pasteur.fr/sante/clre/cadrecnr/rage/rage-actualites.html). Our analysis of the behavior patterns of patients consulting ARMC, and the RPEP and ARIG prescribed to them between 1989 and 2006 was based on those annual data.

Among the 74 French ARMC, 56 systematically entered their data into the NRCR database between 2000 and 2005. The following statistical analysis is based on the exhaustive weekly information provided by these 56 ARMC. The ARMC network also constitutes an effective communication infrastructure coordinated by the NRCR, including conference calls and regular exchanges of information via the internet. When rabies is suspected in a human, biological specimens are sent to the NRCR.

### Newspaper coverage

Articles on rabies-related news published in three major national daily newspapers, *Le Monde*, *Le Figaro* and *Libération*, were retrieved from the French Association for Auditing Media Circulation: an on-line service: http://www.factiva.fr.

### Statistical analysis

Weekly numbers of patients consulting at ARMC, as a function of the date each was in contact with a potentially rabid animal, were used to construct times series. Autoregressive moving average (ARMA) [14] modeling was used to determine the significance of event-associated modification of ARMC weekly patient numbers and its duration. Because several known events could have affected the series, a step-by-step procedure was undertaken [15], [16]. Before the onset of event #2, trend and/or seasonality were estimated and removed, so that the time series was obtained in a stationary mode and, autoregressive integrated moving-average (ARIMA) modeling was done using Box–Jenkins procedure from SAS/ETS [17]). The model was then used to predict ARMC consultations and their 95% confidence intervals (95% CI).

An event was considered to have an impact when the number of consultations during 2 consecutive weeks exceeded the upper 95% CI. Observed values were then replaced by forecasts, to obtain analyses of the subsequent weeks. Similarly, 2 consecutive weeks within the 95% CI defined the end of the event's impact period. Relative differences between observed and predicted values were calculated. For impacting events, the number of cases attributed to the event (NCAE) was estimated by subtracting the prediction from the observed data during the impact period. An increase rate (IR) was then calculated as the ratio of the NCAE/number predicted for the impact period.

With the aim of evaluating potential repercussions of an identified event impacting on RPEP prescriptions, two other time series were investigated: the weekly RPEP rate, defined as the number of RPEP prescribed/the number of consulting ARMC patients, e.g. rabies vaccine with or without ARIG; and the weekly ARIG rate, corresponding to the ratio of the number of ARIG/the number of consulting ARMC patients. During the period associated with modified ARMC weekly numbers, weekly RPEP and ARIG rates and mean numbers of consultations were analyzed using regression with autocorrelated errors to account for the regression residuals (ARIMA procedure).

To explore whether care provided by the ARMC might be influenced by experience in previous French endemic enzootic areas, we divided the country into three areas based on the French administrative regions: area 1, the former enzootic rabies-infected–fox region from 1968 to 1998; area 2, a region that has always remained rabies-free, and area 3, the region where event #6 occurred (Figure 2).

All analyses were performed using R (www.r-project.org) and SAS software.

## Results

**Figure 3:**
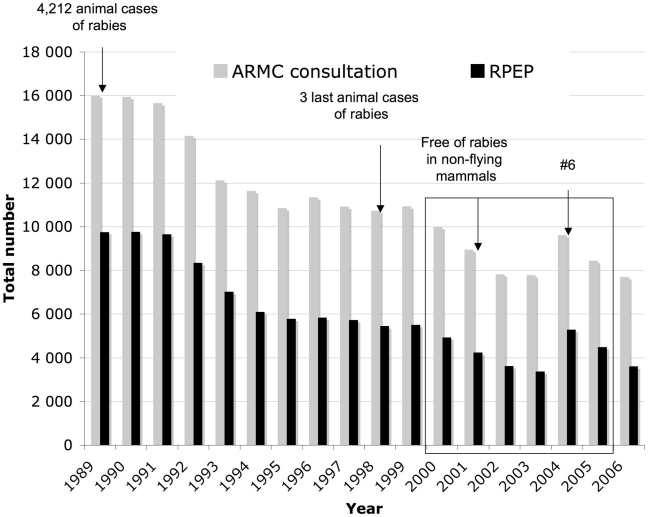
Rabies-exposure notifications to ARMC and numbers of RPEP prescribed to exposed patients in France, 1989–2006. These data are from the annual NRCR report (http://www.pasteur.fr/sante/clre/cadrecnr/rage/rage-actualites.html).

After the reintroduction of rabies into France in 1968, the number of rabid animal cases increased to reach a maximum of 4,212 cases in 1989 [18], followed rapidly by a maximum of 9,763 RPEP prescribed for 15,948 patients consulting at ARMC recorded in 1990 (Figure 3). In 2001, France was declared free of rabies in non-flying terrestrial mammals based on OIE criteria [19] and, as a consequence, the number of patients consulting ARMC and receiving RPEP began to decline progressively to respective minima of 7,788 and 3,378 in 2003 (Figure 3). However, the numbers of patients consulting at ARMC and given RPEP suddenly rose in 2004. Therefore, 2000–2005 data were further investigated using ARIMA modeling to describe in greater detail the trends observed.

Between 1 January 2000 (week 1) and 31 December (week 312) 2005, five rabid dogs illegally imported from Morocco and one rabies-infected human from Gabon were detected in France. During the period examined, the first event #1 dog (5 months old) was confirmed as being rabid in May 2001 (week 74) and the second, event #2 dog (3 months old) in September 2002 (week 139); they entered France from Morocco, 2 months and 2 weeks before their deaths, respectively. The human case (event #3) was a 5-year-old boy, who traveled from Gabon and died 2 months later, in October 2003 (week 199) [20]. Event #4, #5 and #6 dogs were diagnosed as being rabid, respectively, in February 2004 (week 213), May 2004 (week 229), and August 2004 (week 243) [21]. Event #6 was a 4-month-old puppy, illegally imported by car from Morocco to Bordeaux, France, via Spain, who died of rabies in August 2004 (week 243); he was not officially vaccinated.

**Figure 4:**
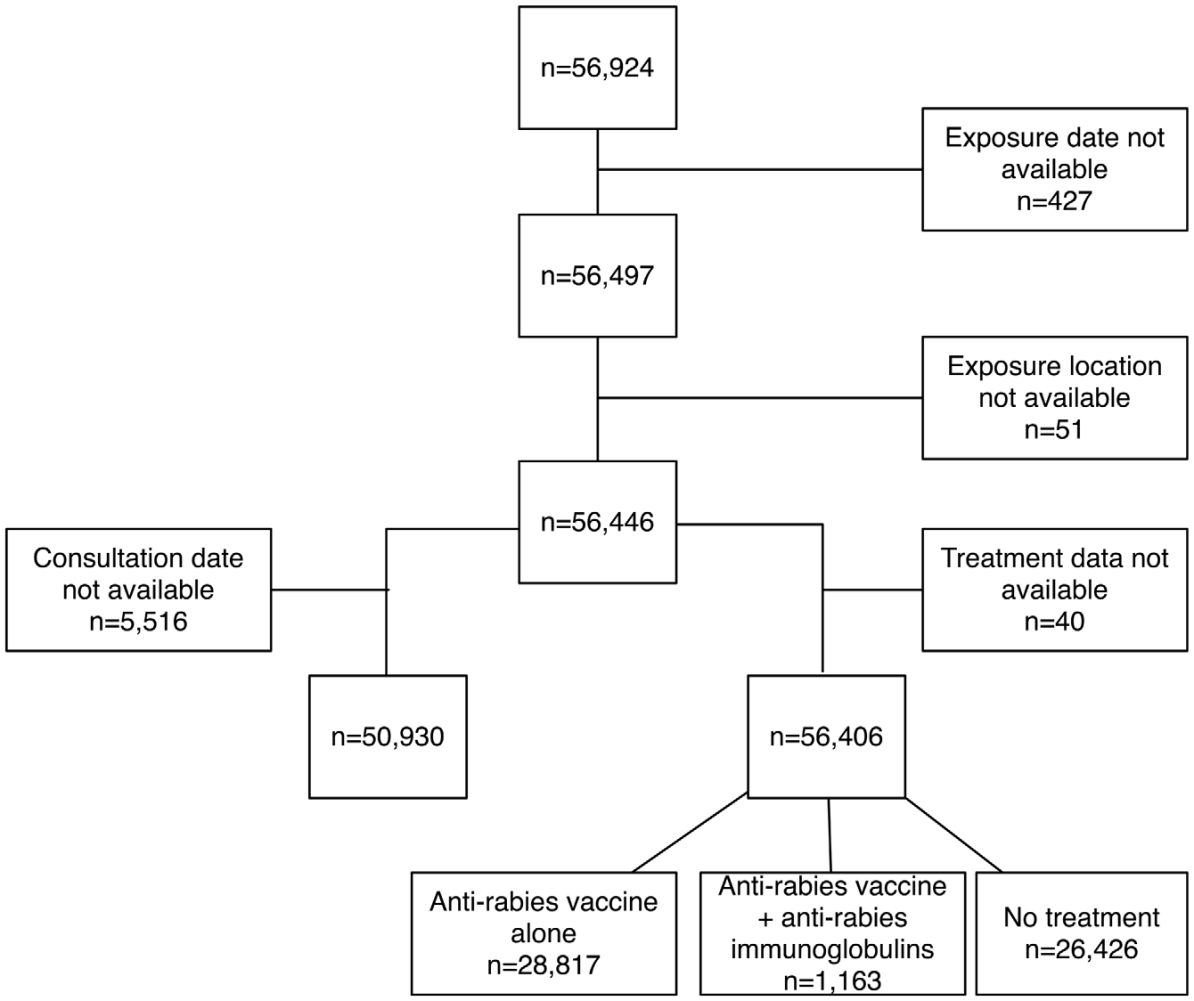
Flow chart of human data used in the analysis.

**Figure 5:**
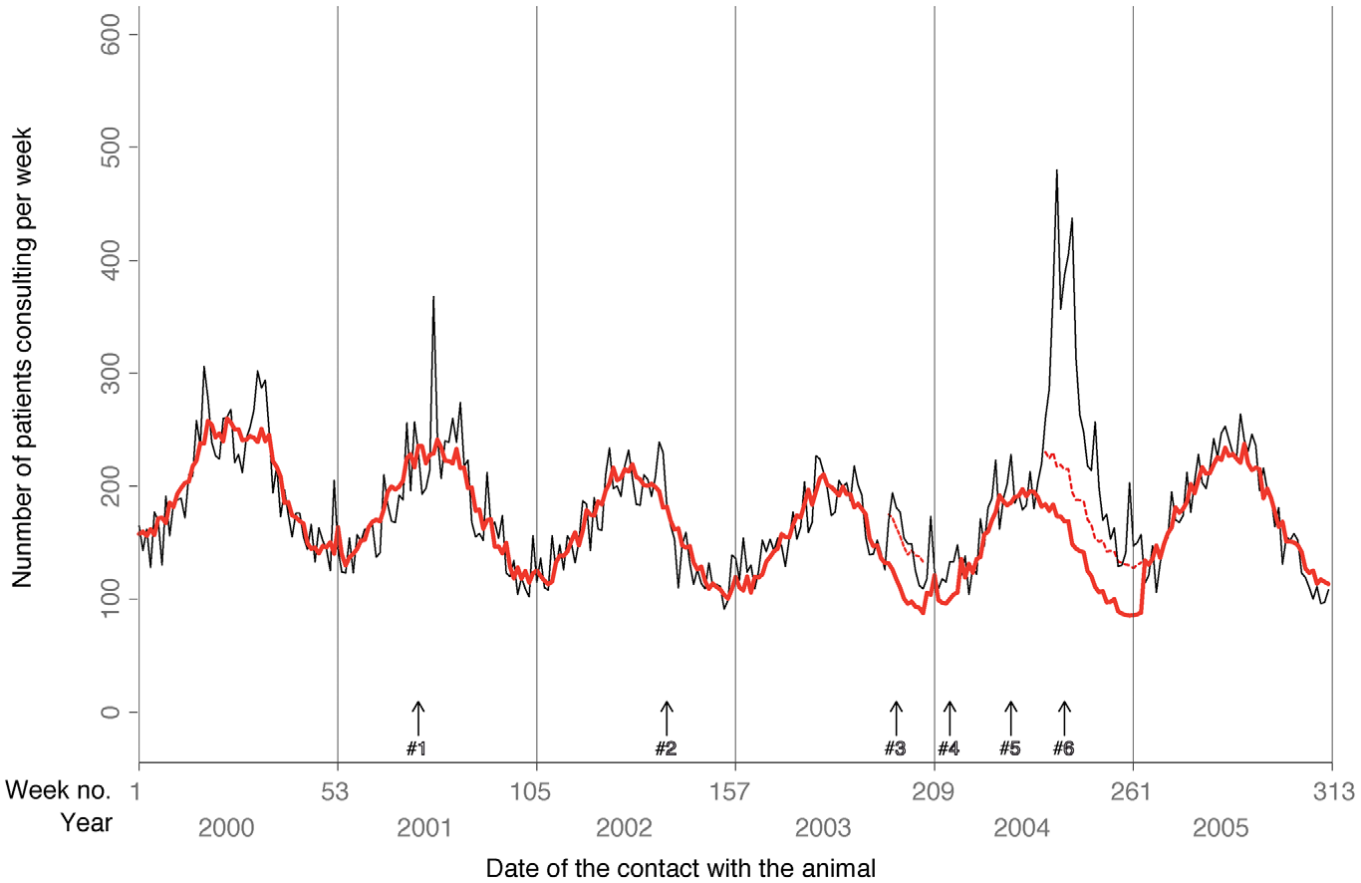
Weekly numbers of notified human contacts with animals that led to a consultation at an ARMC. This figure illustrates the behaviors associated with notified exposures at ARMC (*n* = 56,446). The model combines a forecasting ARIMA model for 2000–2004. Events #1, #2, #4, #5 and #6 correspond to illegal importations of rabid dogs from Morocco, while event #3 was an imported human case from Gabon. Event #1 could not been analyzed because the duration of observations preceding the event was too short to implement ARIMA modeling. The solid black line traces patients' ARMC consultations; the thick red line corresponds to the step-by-step modeling prediction of those consultations; the dashed red lines for event #3 and #6 represent the upper 95% CI. Note the increased consultation rates for these events, especially #6.

Between 1 January 2000 and 31 December 2005, 56,924 rabies-exposed individuals in France (all patients exposed abroad were excluded from the analysis) consulted in an ARMC, among whom 56,446 had valid exposure dates and bite/contact locations. Among them, 50,930 had valid consultation dates and 56,406 had valid treatment information (Figure 4). Because the data presented 52-week seasonality, the time preceding event #1 was too short to be analyzed. In such a case, Box and Jenkins recommend using at least two seasonality periods to calibrate the model [14]. Data analyses concerning events #1, #2, #4 and #5, corresponding to rabid dog importations, were simple and rapidly done, as these dogs had had no known contact with animals and humans other than their owners during their communicable risk periods. As a consequence, events #2, #4 and #5 were not reported in the major national newspapers and were not associated with any significant increase of ARMC activity. In contrast, events #3 and #6 were reported in 6 and 54 published articles retained for this study, respectively, and significantly affected the numbers of patients consulting at an ARMC (Figure 5).

Until event #3 (October 2003), the weekly number of patients consulting an ARMC declined significantly (slope = −0.34; *p*<0.0001), with 52-week seasonality that peaked during the summer (Figure 5). In October 2003, the weekly number of ARMC patients was significantly higher than the predicted number during the 6 weeks surrounding event #3 (weeks 198–203), with an estimated NCAE of 355 (IR = 54.7%, 95% CI = 30.0–83.0). Furthermore, event #3 was followed by a significant flattening of the decreasing slope of ARMC activity (−0.23 versus −0.34; *p* = 0.0003). No RPEP- or ARIG-rate modification associated with event #3 was observed.

In the summer of 2004 (event #6), the weekly number of ARMC patients differed significantly from the predicted number during the 26 weeks surrounding it (weeks 238–263). The total 26-week number of additional ARMC patient load was estimated at 2,928 (IR = 84.0%, 95% CI = 57.0–123.3) over the model predicted 3,486 (Figure 5). During that period, the observed mean RPEP and ARIG rates were significantly higher than those recorded during the period preceding event #6, IR = 19.7% and 43.4%, respectively (Table 1).

**Table 1:** Evolution of rabies post-exposure prophylaxis and antirabies immunoglobulin prescription rates (per 100 people), between 2000 and 2005.

Prescription rates	Before event #6* (Weeks 1–237)	During event #6 (Weeks 238–263)	*p*-Value†	After event #6 (Weeks 264–312)	*p*-Value†
RPEP	50.2 [48.9–51.4]‡	62.5 [59.0–66.0]	<0.0001	58.6 [55.6–61.5]	<0.0001
ARIG‡	1.5 [1.4–1.7]	3.3 [2.7–3.8]	<0.0001	3.53 [3.10–3.97]	<0.0001

*Reference period.

†Compared to the reference period.

‡Values are expressed as mean % (95% CI).

The slopes of the ARMC-consultation decline after week 263 and before week 238 were estimated at −0.12 and −0.23, respectively; *p*<0.001. Surprisingly, between weeks 264 and 312, the mean RPEP rate remained persistently and significantly higher than before the reference period, as did the ARIG rate, which was more than two-fold higher than before week 237 (Table 1).

**Figure 6:**
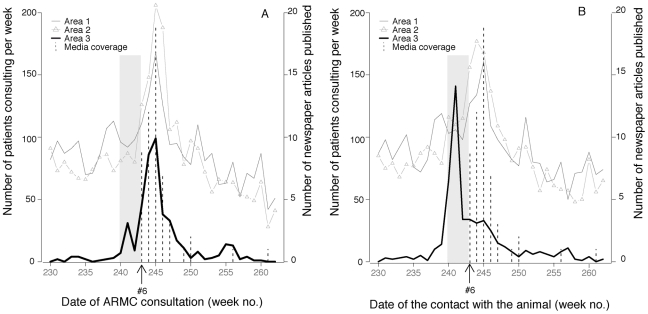
Comparison of the different behaviors observed in the different geographical areas. Keep in mind that event #6 occurred in area 3. The grey band corresponds to the rabid dog's infectious period (weeks 240–242).

The increased number of patients consulting at an ARMC in response to the newspaper articles concerning event #6 peaked at the same time as the media coverage in the three different French areas defined according to their rabies experience (Figure 6A). In area 3, the exposure dates reported by ARMC patients corresponded to the risk period coinciding with the dog's movements and infectivity, whereas in areas 1 and 2, patients reported exposure dates more compatible with newspaper coverage than with the risk period (Figure 6B).

## Discussion

France progressively eliminated rabies in foxes and became rabies-free for indigenous non-flying terrestrial mammals in 2001 [19]. Consequently, use of public health facilities dedicated to the disease decreased steadily from 1990 until 2003, suggesting a continuous impact of rabies elimination on related public health resources and expenses. However, the very mild decline of the 2000–2003 slope probably reflects the difficulties in convincing the public and adapting medical practice to the changing risk. Although elimination of rabies in foxes reduced the number of rabid pets and other domestic animals, and thus exposure to rabies, pet bites continue. Importation of rabid animals and infected travelers returning from abroad also regularly challenge the French public health organization of rabies control. Therefore, the number of RPEP prescriptions and the associated costs will not decline significantly until there is adequate assurance that the probability of a pet being rabid is sufficiently low that such therapy is not warranted, even when the pet's status cannot be verified [22], [23], [24]. Regardless of potential French specificities, public health decision-makers are obliged to consider such potential events and their ensuing demand on medical community resources when attempting to predict and maintain the efficacy of rabies-control policies even in rabies-free countries [24]–[28].

Among the six rabies events occurring during 2000–2005 in France, only two significantly affected ARMC activities and RPEP rates. The human case imported from Gabon in 2003 (event #3) was associated with enhanced ARMC activity during a brief period and also changed ARMC's declining activity, which had been observed since 2000. The boy's demise was reported 6 times in the newspapers, further confirming that “death makes news” for rare and acute diseases [29]. In contrast, the illegally imported rabid dog from Morocco in August 2004 (event #6) had a significant and rapid impact on rabies public health resources. Indeed, the critical shortage of prophylactic drugs resulted from the 84% IR of patients consulting at an ARMC with a 62.5% RPEP rate for those patients over 26 weeks. This influx explains the bottleneck observed in ARMC. Similarly, laboratory rabies-diagnosis workload for animals increased by >40% during the same period (data not shown).

To comply with the threatened shortage of RPEP and ARIG due to the cumulative effect of enhanced patient influx and their more frequent prescriptions, a specific communication strategy was established for the ARMC network to provide information concerning the evolution of the epidemiological situation and to recall the indications of RPEP. This information was disseminated via the websites of the NRCR, the Ministry of Health (MOH), the National Institute for Health Surveillance and the Ministry of Agriculture, which were regularly updated as of 28 August, fax on 2 September, and phone conferences on 3 and 9 September. To complete this plan, temporary licensing of a multidose vaccine (Verorab, Sanofi Pasteur) was accorded and ARIG injections were postponed, as necessary, in accordance with WHO guidelines [12]. Unfortunately, it was not feasible to quantitatively analyze the extent of that adaptation. However, RPEP and ARIG never became completely unavailable. Notably, the risk of a potential ARIG shortage in the event of an unplanned increase of demand or a limitation of supply is shared by many countries in Europe and on other continents [30], [31].

Compared to similar events occurring during 2000–2005 in France, event #6 has several particularities. While only restricted contacts with humans (owners, neighbors…) were suspected for cases #2, #4 and #5, the event #6 dog traveled through southwestern France during the communicable risk period, and had been roaming unleashed at three large summer music festivals, each with at least 10,000–20,000 participants [21]. According to immediate inquiries made by veterinary and medical services, this trajectory potentially led to extensive contacts between the rabid dog and humans and animals.

Therefore, the public health authorities' concern triggered extensive media alerts. First, the MOH wanted to identify and contact each individual with confirmed contact with the event #6 dog. National and local authorities coordinated several news conferences and newspaper reports to inform the French population about the risk and recommendations concerning errant dogs in general, and how to react to potential exposure to a rabid dog. A European-wide alert was launched through the European warning and response system. Second, beginning in early September 2004, this intensive communication frenzy of 54 newspaper articles heightened public awareness of the rabies risk. Third, additional public concern might also have been heightened by controversies surrounding the crisis management. Notably, event #6 occurred just before the annual opening of hunting season, in a strongly traditional hunting region. An initial decision was made to forbid hunting with dogs in the counties where the rabid dog had traveled during his infectious period. That restriction led to a passionate public debate, angering hunters and ending with hunting organizations successfully blocking the ban. Fourth, public health authorities decided to eradicate free-roaming dogs. Finally, press releases issued by the Minister of Rural Affairs and the MOH were contradictory concerning the implementation of mandatory antirabies vaccination of dogs and cats.

The constant media attention drawn by these different players during event #6 may have contributed to enhancing the sense of rabies risk, thereby prompting people to associate dog bites with rabies and to consult at an ARMC [3], [32], [33]. Furthermore, public health crises, e.g. that generated by severe acute respiratory syndrome, demonstrated how conflicting messages can create confusion and uncertainty in both the media and the general public [21]. However, this event #6 newspaper coverage, initiated and promoted by public health authorities, reached its primary and immediate objective, e.g., no secondary dog or human rabies case was reported following the dog's arrival in France. Eight of the 13 identified individuals, who had been exposed to the rabid dog, were located and contacted and 49 dogs and 8 cats identified as having been in direct contact with the event #6 dog were killed. We would have expected this unusual news coverage of a rabies event to have raised public awareness about the risks of illegally importing animals from endemic countries. Between 2000 and 2005, France was the only rabies-free European country to have so many imported cases. Unfortunately, in 2007–2008, two new dog-importation episodes were reported in France, clearly illustrating the short persistence of this type of information disseminated to the public. Because of one of these events, France lost its rabies-free status according to OIE criteria in 2008.

We only examined national newspaper stories available in Factiva but not local newspaper reporting or television, radio and internet stories, and, thus, probably underestimated the global coverage of these episodes. In response to national newspaper coverage, people who are far from the event location can become concerned and start taking precautions as if they were in the affected area [3], [4], [32]. This phenomenon is particularly well illustrated by event #6, for which exposure dates reported by patients consulting at AMRC in areas 1 and 2 corresponded to the period of newspaper coverage rather than to the risk-of-transmission period during the dog's movements.

Lastly, long-term modifications of ARMC activity and RPEP- and ARIG-prescription rates were observed. In particular, 2005 RPEP and ARIG rates (ARIMA study herein) and even those for 2006 had not yet returned to 2003 levels. This finding strongly suggests a persistent and unjustified heightened perception of the risk by individuals and physicians, even those specialized in rabies treatment, and this despite regular information provided by the NRCR to the ARMC network and a rapidly controlled situation with no recorded secondary animal and human cases during the following 2 years.

In conclusion, event #6 and its associated national newspaper coverage profoundly perturbed health services, with excessive consulting at ARMC and durably increased antirabies drug rates for several months, along with more animal diagnostic testing. This crisis highlighted a lack of experienced manpower and insufficient vaccine stocks. Outbreaks of emerging and/or deadly infections, like severe acute respiratory syndrome [34]–[38], anthrax [39], [40] and rabies (herein), have shown that media messages dramatically influence both the public's and health-care workers' perceptions of the risk with potential implications for health-care resources. Our observations underscore to what extent, under such circumstances, public health decision-makers have to anticipate the depth and scope of potential consequences of emerging or reemerging infectious diseases and their related press communications, and the need to prepare appropriate responses to keep the public health organization effective. It also illustrated that, despite communication efforts implemented by the French public health authorities and messages released through the ARMC network, long-term modifications of ARMC activities and prescriptions were observed, further emphasizing that a post-crisis communication strategy is essential.

## Supporting Information

Table S1 Case-report form for human exposure to rabies used in France. Since 2006, collection and dissemination of information are made by filling out questionnaires available at a centralized online site named Voozanoo (http://www2.voozanoo.net/tiki-index.php?page=What%27sVoozanoo).
(0.07 MB DOC)
