# Supplementary material for: Imported Episodic Rabies Increases Patient Demand for and Physician Delivery of Antirabies Prophylaxis
Source: PLoS Negl Trop Dis. 2010 Jun 22;4(6):e723. doi: 10.1371/journal.pntd.0000723 (PMC2889823; doi:10.1371/journal.pntd.0000723)
Supplement: Table S1 — Case-report form for human exposure to rabies used in France. Since 2006, collection and dissemination of information are made by filling out questionnaires available at a centralized online site named Voozanoo (http://www2.voozanoo.net/tiki-index.php?page=What%27sVoozanoo). (0.07 MB DOC) [file pntd.0000723.s001.doc]

**Supporting information**

**Table S1. Case-report form for human exposure to rabies used in France**. Since 2006, collection and dissemination of information are made by filling out questionnaires available at a centralized online site named Voozanoo (http://www2.voozanoo.net/tiki-index.php?page=What%27s+Voozanoo).

**National Reference Center for Rabies** **Monthly report on rabies prophylaxis given humans**

Institut Pasteur

25, rue du Docteur Roux Antirabies medical center:............................................

75724 Paris cedex 15 Month: ............... Year: ..................

France

| No. | Age | Sex | Exposure | | | | | Animal | | | | Rabies post-exposure prophylaxis (RPEP) | | | | | | Comments |
| --- | --- | --- | --- | --- | --- | --- | --- | --- | --- | --- | --- | --- | --- | --- | --- | --- | --- | --- |
|  |  | M F U | Date | City | District | Country | Type | Species | Result of investigation | | | RPEP | Interval | ARIG | Vaccine | Compliance | Reaction |  |
|  |  |  |  |  |  |  | 3 2 1 |  | Negative | Positive | U | Y N P |  | H E N |  | C S A | N L G |  |
|  |  |  |  |  |  |  |  |  | Clin Lab | Clin Lab |  |  |  |  |  |  |  |  |
|  |  |  |  |  |  |  |  |  |  |  |  |  |  |  |  |  |  |  |
|  |  |  |  |  |  |  |  |  |  |  |  |  |  |  |  |  |  |  |
|  |  |  |  |  |  |  |  |  |  |  |  |  |  |  |  |  |  |  |
|  |  |  |  |  |  |  |  |  |  |  |  |  |  |  |  |  |  |  |

**Instructions for filling out the form**

Sex: M: male

F: female

U: unknown

Exposure

Date: date of exposure

Type: type of exposure according to WHO classification

Animal

Species: animal species responsible for exposure

Result of investigation: Clin: result of veterinary surveillance

Lab: result of the laboratory diagnosis

U: unknown

RPEP: Y: Yes

N: No

P: passage (someone who had already started RPEP in another ARMC)

Interval: number of days between exposure and start of RPEP

ARIG (antirabies immunoglobulin): H: human origin

E: equine origin

N: None

Vaccine: type of vaccine used

Compliance: C: RPEP completed

S: RPEP stopped by the physicians

A: Abandoned

Reaction: N: None

L: Local

G: General
